# Involvement of miRNAs in the Differentiation of Human Glioblastoma Multiforme Stem-Like Cells

**DOI:** 10.1371/journal.pone.0077098

**Published:** 2013-10-14

**Authors:** Beatriz Aldaz, Ainara Sagardoy, Lorena Nogueira, Elizabeth Guruceaga, Lara Grande, Jason T. Huse, Maria A. Aznar, Ricardo Díez-Valle, Sonia Tejada-Solís, Marta M. Alonso, Jose L. Fernandez-Luna, Jose A. Martinez-Climent, Raquel Malumbres

**Affiliations:** 1 Division of Oncology, Center for Applied Medical Research (CIMA), University of Navarra, Pamplona, Spain; 2 Molecular Genetics Unit, Hospital Universitario Marques de Valdecilla and Instituto de Formacion e Investigacion Marques de Valdecilla (IFIMAV), Santander, Spain; 3 Unit of Proteomics, Genomics and Bioinformatics, Center for Applied Medical Research (CIMA), University of Navarra, Pamplona, Spain; 4 Department of Pathology, Memorial Sloan-Kettering Cancer Center, New York, New York, United States of America; 5 Human Oncology and Pathogenesis Program, Memorial Sloan-Kettering Cancer Center, New York, New York, United States of America; 6 Department of Neurosurgery, Clínica Universidad de Navarra, Pamplona, Spain; University of Florida, United States of America

## Abstract

Glioblastoma multiforme (GBM)-initiating cells (GICs) represent a tumor subpopulation with neural stem cell-like properties that is responsible for the development, progression and therapeutic resistance of human GBM. We have recently shown that blockade of NFκB pathway promotes terminal differentiation and senescence of GICs both *in vitro* and *in vivo*, indicating that induction of differentiation may be a potential therapeutic strategy for GBM. MicroRNAs have been implicated in the pathogenesis of GBM, but a high-throughput analysis of their role in GIC differentiation has not been reported. We have established human GIC cell lines that can be efficiently differentiated into cells expressing astrocytic and neuronal lineage markers. Using this *in vitro* system, a microarray-based high-throughput analysis to determine global expression changes of microRNAs during differentiation of GICs was performed. A number of changes in the levels of microRNAs were detected in differentiating GICs, including over-expression of hsa-miR-21, hsa-miR-29a, hsa-miR-29b, hsa-miR-221 and hsa-miR-222, and down-regulation of hsa-miR-93 and hsa-miR-106a. Functional studies showed that miR-21 over-expression in GICs induced comparable cell differentiation features and targeted *SPRY1* mRNA, which encodes for a negative regulator of neural stem-cell differentiation. In addition, miR-221 and miR-222 inhibition in differentiated cells restored the expression of stem cell markers while reducing differentiation markers. Finally, miR-29a and miR-29b targeted *MCL1* mRNA in GICs and increased apoptosis. Our study uncovers the microRNA dynamic expression changes occurring during differentiation of GICs, and identifies miR-21 and miR-221/222 as key regulators of this process.

## Introduction

Glioblastoma multiforme (GBM) is the highest grade (IV) astrocytoma and the most common glioma, accounting for ~40% of all primary brain tumors of the central nervous system (CNS) [1]. GBM is one of the most aggressive tumors. Patients usually have a median overall survival of 12-15 months, due to the high rate of tumor recurrence despite surgical tumor removal and radio-chemotherapy, which highlights the need for more effective therapies [2]. It has been proposed that gliomagenesis initiates in adult neural stem cells or neural precursors that undergo transformation into GBM-initiating cells (GICs), which display a stem cell-like behavior [3]. GICs are able to self-renew, express stem cell markers such as CD133 and Nestin, and can generate and propagate tumors in immunodeficient mice [3-5]. In addition, GICs are highly resistant to current therapies, possibly explaining the frequent tumor relapses [6]. Of note, GICs can be induced to differentiate into mature cells of the main CNS lineages, which lose their stem cell behavior and become more sensitive to certain therapies [3]. As representative examples, differentiation of CD133^+^ GBM cells with bone morphogenetic protein 4 (BMP4) or using an all-trans retinoic acid (ATRA)-based treatment led to inhibition of the tumorigenic potential of these cells and resulted in retardation of GBM growth in mice, as well as in sensitizing cells to radiation and BCNU chemotherapy in the case of ATRA [7,8]. Furthermore, our group recently discovered that blockade of NFκB pathway promotes terminal differentiation and senescence of GICs both *in vitro* and *in vivo* [9]. All these data suggest that induction of differentiation may be a potential therapeutic strategy for GBM.

MicroRNAs (miRNAs) are small non-coding RNAs (21-23 nucleotides long) that bind to specific sites in the 3´-UTR of their target mRNAs by partial complementarity, subsequently inducing their degradation and/or the inhibition of their translation [10]. miRNAs play a number of different roles in the regulation of stem cell biology, differentiation, and cell identity [10]. For example, miRNAs have been implicated in the transition from neural stem/precursor cells to differentiated neurons [11]. In addition, miRNAs are key players in tumor development, including GBM [12]. Several miRNAs display deregulated expression in GBM samples, and some of them have been shown to regulate differentiation of GICs into mature neural-like cells [13,14]. Accordingly, the use of interfering RNAs aiming to induce GIC differentiation may represent a promising therapeutic approach in malignant gliomas [15]. 

However, a global analysis of miRNA expression changes occurring during GIC differentiation has not been performed yet. We have recently established several human GIC lines that can be efficiently differentiated into cells expressing astrocytic and neuronal lineage markers *in vitro* [9,16]. Using this system, here we performed a microarray-based high-throughput miRNA expression analysis to uncover the dynamic expression changes of miRNAs during GIC differentiation. Our study identified several miRNA and their potential target genes that may play a role in this process. 

## Materials and Methods

### Ethic Statements

Human glioblastoma samples were obtained after written consent for the research use of the specimens was provided by all patients. These procedures were approved by the institutional review boards of Hospital Universitario Marques de Valdecilla and Clínica Universidad de Navarra. The study involves the use of completely anonymized specimens.

The xenografts experiments in mice were performed at the Animal Core Facilities of the Center for Applied Medical Research (University of Navarra) after approval by the University of Navarra Animal Ethics Committee. To avoid suffering, the animals were anesthetized with i.p. ketamine-xylazine 3:1 for surgical procedures and with continuous inhalation of 2% isoflurane during PET.

### Cell line culture

The U87MG GBM cell line (ATCC HTB-14) was cultured in DMEM (Invitrogen) supplemented with 10% FBS (Gibco) and 2% penicillin/streptomycin (BioWhittaker, Lonza).

### Primary tumor neurosphere (NS) cultures

NS cultures were derived from five biopsies obtained from patients diagnosed of GBM, as previously described [9]. Briefly, surgical samples were washed, followed by mechanical dissociation and enzymatic digestion. Tumor cells were then cultured in serum-free DMEM/F12 medium (Invitrogen) containing 20 ng/ml human recombinant EGF (Sigma), 20 ng/ml bFGF (Sigma) and 2% B-27 supplement (Invitrogen). Primary neurospheres were detected within the first two weeks of culture and subsequently dissociated every 3-4 days to facilitate cell growth. To promote differentiation, neurospheres were cultured in the same medium without B-27 supplement and 10% fetal bovine serum (FBS) was added. 

### Self-renewal assessment

Clonogenic and limiting dilution assays were performed as previously described [17] with minor modifications. Two different *in vitro* self-renewal assays were performed: the clonal dilution assay, measured as the mean percentage of wells containing at least one NS after seeding the cells at a clonal dilution (1 cell/well) and culturing them for 10 days, and the limiting dilution assay, which indicates the number of cells from a primary NS that are needed to form a secondary NS. For this experiment, primary neurosphere cultures were dissociated and seeded at dilutions that ranged from 200 cells/well to 1 cell/well. After 7 days of culture, the percentage of wells not containing spheres was plotted against the number of cells seeded per well to calculate the corresponding regression lines. The intersection of these lines with the X-axis corresponds to the number of cells needed to form at least one NS. Cells from the GBM cell line U87MG, not enriched in GICs, were grown in NS culture medium in parallel and used as negative control for both assays. 

### *In vivo* experiments

Xenograft experiments in mice were performed as previously described [9]. Briefly, one million NS cells were injected into the brain (caudate putamen region) of anesthetized 6–8-week-old female BALB/c-Rag2^-/-^-IL2γc^-/-^ using a microsyringe held in a stereotactic device (Kopf Instruments). Eight weeks after injection, xenografted mice were monitored for tumor metabolic activity by microPET in a dedicated small-animal Philips Mosaic tomograph (Philips). Anesthetized mice were injected with 11C-methionine (20 Mbq) and then placed prone on the PET scanner bed to perform a static acquisition. Maximum standardized uptake value (SUVmax) was calculated for each tumor. For histopathological studies of the tumors, anesthetized animals were perfused with 4% paraformaldehyde and their brains were removed, post-fixed, sectioned and stained with hematoxylin-eosin. Coronal sections (200 mm thick) of the brain at the level of striatum were examined with a stereoscopic microscope for tumor localization. 

### Cell transfection experiments

NSs were disaggregated with Accutase solution (Sigma-Aldrich) and 1-2x10^5^ cells were transfected with 100 nM pre-miRNA or anti-miRNA oligonucleotides specific for miR-21, miR-29a, miR-29b, miR-221 and miR-222 or pre/anti-miRNA negative controls 1 (Ambion) using Nanojuice (Novagen) following the manufacturer´s instructions. For luciferase reporter experiments, the cells were transfected in triplicate with the appropriate 3´-UTR-luciferase construct (0.2 ng/µl) and the corresponding pre-miRNA (100 nM) using Nanojuice (Novagen). To produce the 3´-UTR-luciferase constructs, the 3′-UTR regions of *MCL1* and *SPRY1* were amplified from human genomic DNA using the Phusion High-Fidelity PCR Master Mix (Thermo Scientific) with specific primers (Table S1), PCR products were digested with XhoI and NotI (New England Biolabs) and ligated into the psiCHECK-2 vector (Promega). Putative miRNA binding sites were identified using the PITA algorithm (http://genie.weizmann.ac.il/pubs/mir07/mir07_prediction.html) and mutated using a strategy based on nested PCRs [18] using Phusion™ High-Fidelity PCR Master Mix (Thermo Scientific) and specific primers (Table S1). After 24 hours, luciferase measurements were performed with Dual-Luciferase Reporter Assay System (Promega) in the Berthold LUMAT 9507 luminometer. Each experiment was repeated at least 3 times. Statistical analysis was performed with the unpaired t test for SPRY1 3´-UTR (Gaussian distribution) or the Mann-Whitney test for MCL1 3´-UTR, (data did not fit the Gaussian distribution) using the Holm-Bonferroni correction for multiple comparisons.

### Immunofluorescence microscopy

Cells were assayed for the presence of Nestin, GFAP, TUJ1 and O4 by immunofluorescence as previously described [9]. Briefly, neurospheres were collected on microscope slides by cytospin centrifugation (400 rpm, 1 min) and differentiated cells were grown on *LabTek chamber slides* (Nunc). Cells were then fixed in 4% paraformaldehyde and permeabilized with 0.5% Triton X-100. For immunostaining, cells were incubated overnight with rabbit anti-GFAP (DAKO, Z0334), mouse anti-Nestin (BD Biosciences, 611658), mouse anti-Tuj1 (Sigma, T-8660) or mouse anti-O4 (Millipore, MAB345) antibodies. Texas red-conjugated or fluorescein isothiocyanate (FITC)-conjugated goat anti-rabbit or anti-mouse (Jackson ImmunoResearch) were used as secondary antibodies. Nuclei were counterstained with 4’,6-diamidino-2-phenylindole (DAPI). Images were captured with a 739 CCD camera coupled to an Axio Imager Z1 microscope (Carl Zeiss Inc.) using the Plan-Neofluar 20x/0.50 objective and the Isis Imaging System software (Metasystems). For quantification purposes, FITC-conjugated AffiniPure Goat Anti-Mouse IgG (H+L) and FITC-conjugated AffiniPure Goat Anti-Rabbit IgG (H+L) (both from Jackson ImmunoResearch) were used as secondary antibodies and images were acquired with an Axiocam Mrm camera coupled to an Axioimager M1 fluorescence microscope (Carl Zeiss Inc.), using the Plan-Neofluar 20x/0.50 NA objective and the Axiovision (4.6.3.0) program. Fluorescence was quantified using FIJI plugin (ImageJ V1.46b) built in-house. The plugin packs image processing operations as co-location analysis, filtering and particle counting to automatically measure the mean FITC intensity associated to a cell (located in the proximity of DAPI signals) in the field. 

In addition, cells were assayed for the simultaneous presence of Nestin, GFAP and TUJ1 by triple immunofluorescence as previously described [9], using as primary antibodies mouse anti-Nestin (BD), rabbit anti-GFAP (DAKO) and chicken anti-TUJ1 (Aves labs, TUJ6797987). Alexa fluor 488 goat anti-mouse IgG, Alexa fluor 568 goat anti-rabbit IgG and Alexa fluor 647 goat anti-chicken IgG (Invitrogen) were used as secondary antibodies. Images were acquired with a Leica SP5-II confocal microscope (Leica microsystems) using a 20x/0.7 NA water immersion objective.

### Cell viability and apoptosis assays

In order to test cell viability, 5,000 cells per well were plated in 96-well tissue culture plates 24 hours after transfection with the corresponding pre-miRNAs. Cells were then cultured for 72 hours and cell viability was measured by using the Cell-Titer 96 One Solution Aqueous kit (Promega). To assess apoptosis, 50,000 cells were harvested 96 hours after transfection with the corresponding pre-miRNAs and processed using the Cell Death Detection ELISA^PLUS^ kit (Roche) following the manufacturer´s instructions. All studies were carried out in triplicate wells and at least 3 independent transfection experiments were analyzed.

### Western Blot analysis

Cells were collected 96 hours after transfection and whole-cell lysates were analyzed by Western blot with specific antibodies against MCL1 (Stressgen), SPRY1 (sc-30048; Santa Cruz) and ACTB (β-Actin) (Calbiochem) as previously described [19].

### miRNA and gene expression microarrays

Total RNA from the GBM cell lines G52, G63, G59, GN1C and G97C, at the NS state or after 4 or 14 days of differentiation, was extracted using mirVana miRNA Isolation Kit (Ambion), labeled with Hy3 with the miRCURY Hy3/Hy5 Power labeling kit (Exiqon) and hybridized onto miRNA miRCURY™ LNA Array version 5^th^ Generation (Exiqon) mixed with a pool of all samples labeled with Hy5. These microarrays contain 1891 capture probes complementary to human, mouse, rat, and their related viral sequences from the v.14.0 release of miRBase, as well as human miRPlus™ sequences not yet in miRBase. Data were normalized by Lowess and only miRNAs with expression values over background in more than 50% of the samples of at least one experimental condition were considered for statistical analysis. Three comparisons were performed (4 days vs. NS, 14 days vs. NS and 4 and 14 days of differentiation together vs. NS) using the LIMMA R package [20]. MicroRNAs showing values of B>0 in any of the 3 comparisons and Hy3 raw data values greater than 200 in average were selected for validation and further analysis. Hierarchical clustering of microarray data was used to generate heat maps of expression using Cluster 2.11 and Treeview 1.60 (http://rana.lbl.gov/EisenSoftware.htm) [21].

Gene expression microarray hybridization using the Human Genome U133 Plus 2.0 Array (Affymetrix) was performed for the GBM cell lines G48, G52, G63 and G59 at the NS state and after 4 days of differentiation, as previously described [9]. Both background correction and normalization were performed using the RMA (Robust Multichip Average) algorithm [22]. Probe sets with a log2Ratio of gene expression in the differentiated state to the NS state over 1.0 or below -1.0 in at least 2 samples, and with the same tendency in the rest, were selected as differentially expressed.

The data from both microarray experiments are available in the GEO database (http://www.ncbi.nlm.nih.gov/geo/) under the accession number GSE44843. 

### Quantitative RT-PCR (q-RT-PCR)

Validation of candidate miRNAs was carried out using specific TaqMan MicroRNA assays (Applied Biosystems) for q-RT-PCR. 

To assess the expression of individual genes, RNA was extracted with TRI Reagent (Invitrogen) and q-RT-PCR was performed with FastStart Universal SYBR Green Master (Rox) (Roche Diagnostics) in a 7300 Real Time PCR System (Applied Biosystems) using specific primer pairs to detect the expression of *NES* (^5′^CTTCCCTCAGCTTTCAGGAC^3′^; ^5′^TAAGAAAGGCTGGCACAGGT^3′^), *TUJ1* (^5′^GGCCTGACAATTTCATCTTTGG^3′^; ^5′^TCGCAGTTTTCACACTCCTTC^3′^), *GFAP* (^5′^GCAGAGATGATGGAGCTCAATGACC^3′^; ^5′^GTTTCATCCTGGAGCTTCTGCCTCA^3′^) and *GAPDH* (^5′^AGCCACATCGCTCAGACAC^3′^; ^5′^CCATGTAGTTGAGGTCAATGAA^3′^).

Difference in threshold cycle (ΔCt) was calculated as the subtraction of the Ct corresponding to the housekeeping gene (*GAPDH*) or small RNA (RNU6B) from the Ct of the gene or miRNA of interest for each sample. ΔΔCt was obtained by subtracting the ΔCt corresponding to the NS state to the ΔCt of the cells differentiated during 4 or 14 days. Fold change (FC) was calculated as 2^-ΔΔCt^ for values greater than 1. For values below 1, the symmetric value -1/2^-ΔΔCt^ was calculated. At least three independent experiments with triplicate wells for each qRT-PCR were performed.

### Prediction of potential miRNA targets

miRNA target predictions were extracted from the following public databases: TargetScan v.5.1 (http://www.targetscan.org) [23], PicTar 2006 (http://pictar.mdc-berlin.de) [24], PITA v.6 (http://genie.weizmann.ac.il/pubs/mir07/mir07_data.html) [25], miRanda sept2008 (http://www.microrna.org/microrna/getDownloads.do) [26] and microCosm v.5. (http://www.ebi.ac.uk/enright-srv/microcosm/htdocs/targets/v5/) [27]. A gene list was generated combining down-regulated genes potentially targeted by up-regulated miRNAs and *vice versa*. The functional *in silico* analysis of these genes was performed using Ingenuity Pathway Analysis (IPA) 9.0 (Ingenuity Systems, www.ingenuity.com).

### Statistical analyses

Statistical analyses were performed with GraphPad Prism 4.0b. Variables were tested for fitness to Gaussian distribution with the Shapiro-Wilk test over residual values. Comparisons were performed by means of unpaired t test if the variable fitted to a Gaussian distribution or Mann-Whitney U test if not, correcting for multiple comparisons in both cases according to the Holm-Bonferroni method. 

## Results

### Characterization of the neurosphere cultures obtained from surgical GBM samples

**Figure 1:**
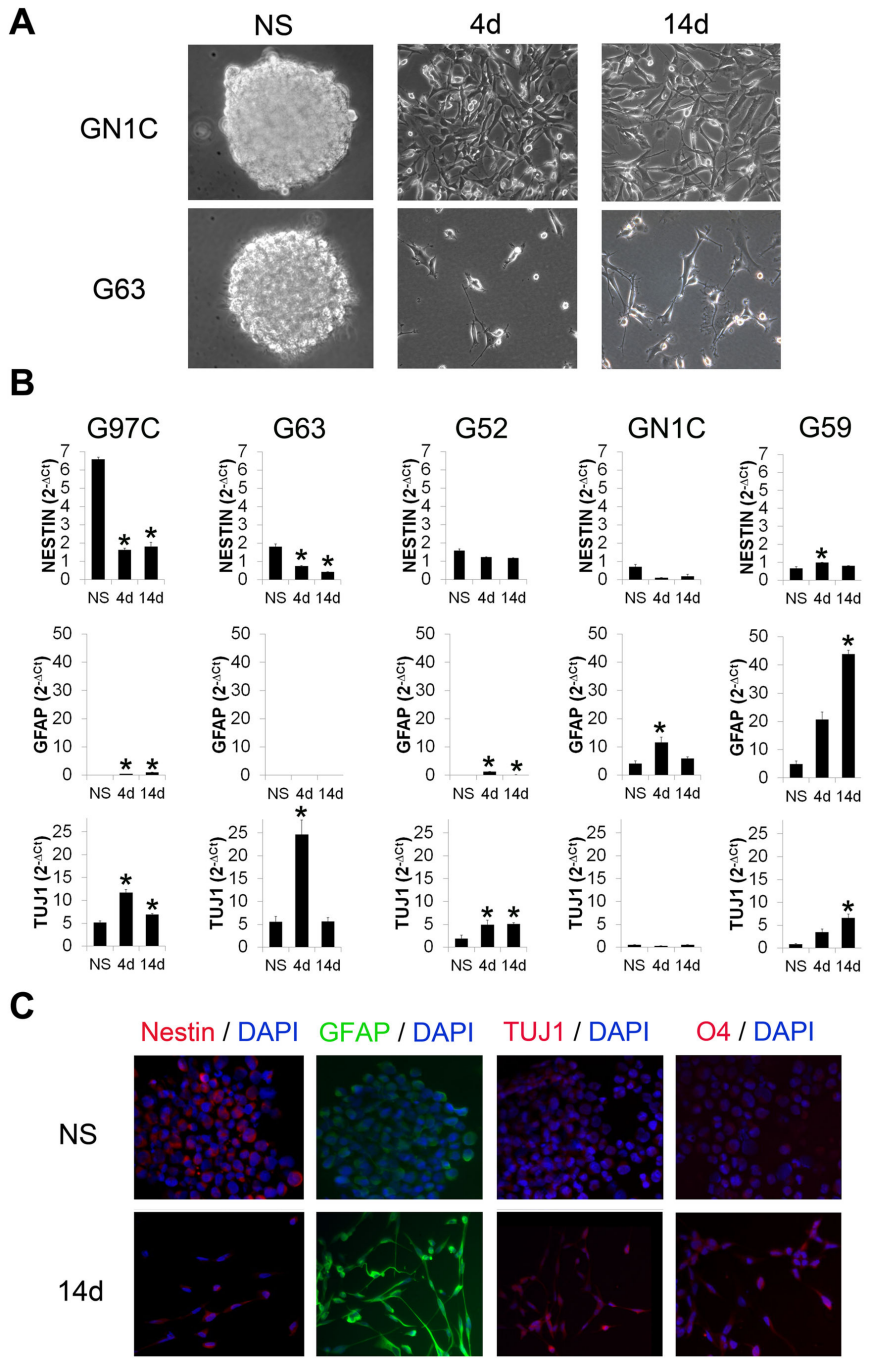
The GBM neurosphere cultures express neuronal and astrocytic differentiation markers upon *in* *vitro* induced differentiation. Morphology of two representative NS cell lines, GN1C and G63, is shown at their basal state of NS (A, left panels) and after induction of their differentiation for 4 (4d) (A, central panels) and 14 days (14d) (A, right panels). All images were captured using an inverted optical microscope Leica DMIRB with 20X magnification. Expression of progenitor (Nestin), astrocytic (GFAP) and neuronal (TUJ1) markers was measured by q-RT-PCR in the NS cell lines G59, G97C, G63, G52 and GN1C (B) at the basal NS state and upon 4 (4d) or 14 (14d) days of induction of differentiation. Data were normalized to *GAPDH* expression as 2^-ΔCt^. * indicates statistical p value <0.05 using unpaired t-test with the Holm-Bonferroni correction for multiple comparisons. Protein expression of Nestin, GFAP and TUJ1, as well as the presence of the oligodendrocytic sulfatide marker O4 (C), were analyzed by immunofluorescence in the GN1C cell line at the NS state (NS) and after 14 days (14d) of differentiation using secondary antibodies conjugated to FITC (green) or Texas Red (red) counterstained with DAPI (blue). Images were acquired at 20X magnification with a 739CCD camera coupled to an Axio Imager Z1 microscope (Carl Zeiss Inc.) using the Isis Imaging System software.

Five cell lines derived from GBM biopsies were cultured in a GIC-propagating medium, where they formed typical spherical structures in suspension known as neurospheres (NS) (Figure 1A, left panels) expressing the neural progenitor cell marker Nestin (Figure 1B-C). Addition of 10% FBS and withdrawal of B-27 supplement induced differentiation of NS cells, which acquired morphological features resembling those of glial and neuronal cells. These included growth in cell monolayers attached to the flask and development of cell protrusions (Figure 1A). In addition, a statistically significant increase in the mRNA levels of GFAP (a protein expressed by cells in the astrocytic lineage) and/or TUJ1 (a β-tubulin protein expressed by cells in the neuronal lineage) was observed (Figure 1B). In contrast, Nestin expression displayed a statistically significant decrease upon differentiation in G97C and G63, as well as a tendency to decrease in G52 and GN1C (Figure 1B). The expression of these markers was also evaluated at protein level by immunofluorescence (Figure 1C). In agreement with mRNA expression levels, GN1C cells showed a decrease of Nestin and an increase in GFAP, while TUJ1 seemed mostly unchanged in this cell line (Figure 1B-C). Differentiation to the oligodendrocytic lineage, determined by immunostaining of O4 sulfatides, was scarce or null (Figure 1C), which is consistent with previously reported data [28]. In order to study whether different cells in the differentiated cultures expressed TUJ1 and GFAP separately or both markers were co-expressed in the same cells, we performed triple immunofluorescence staining for GFAP, TUJ1 and Nestin. These experiments revealed that differentiating GICs co-expressed GFAP and TUJ1, while Nestin expression was markedly reduced (Figure S1A). These results show that the NS cell lines can be efficiently differentiated to cells expressing both neuronal and astrocytic markers.

**Figure 2:**
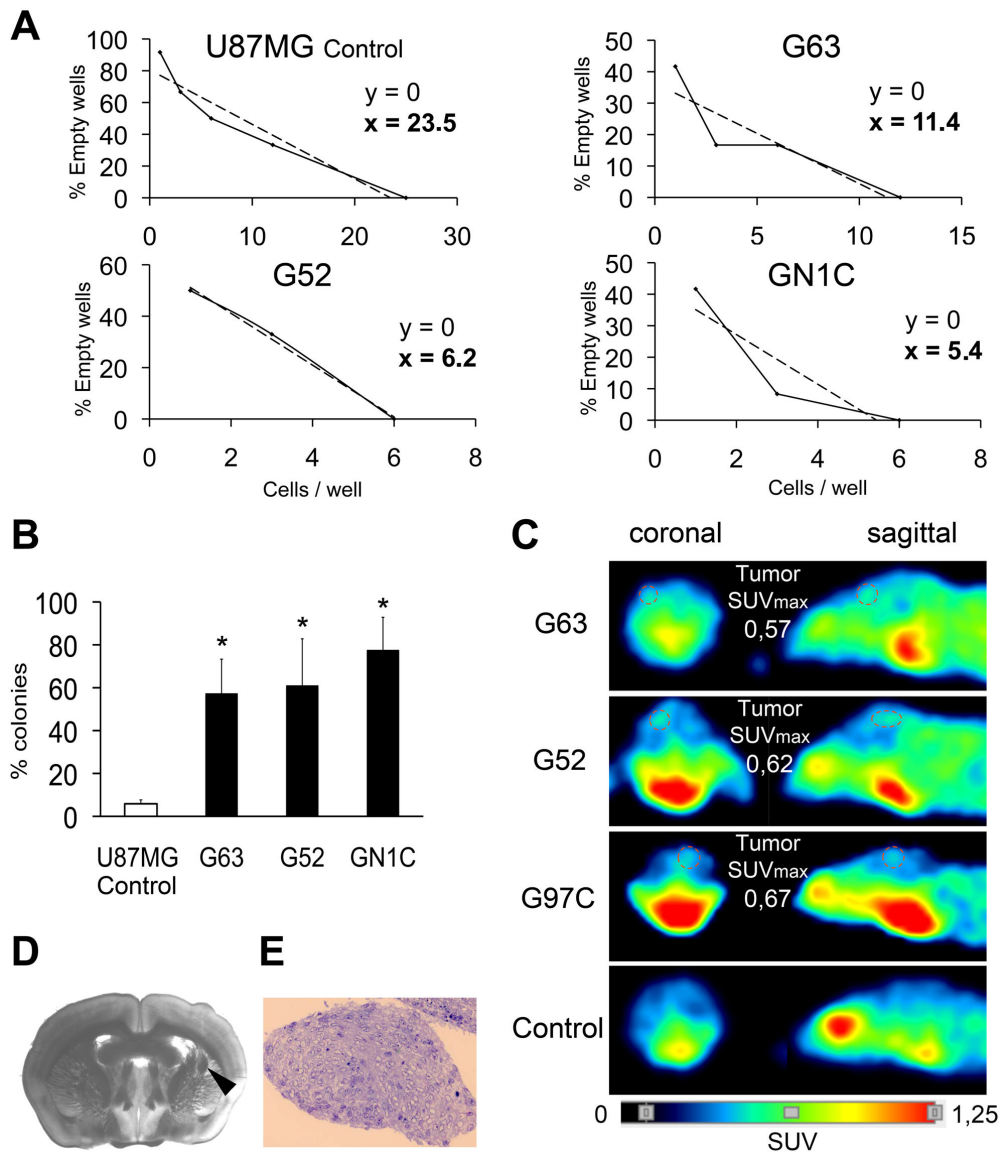
The NS cultures are enriched in GICs when compared to the glioblastoma cell line U87MG. *In* *vitro* self-renewal limiting dilution assays, performed in 12 wells per dilution in triplicate experiments (A), and clonogenic assays, performed in quadruplicate 96-well plates (B) for three NS cell lines: G63, G52 and GN1C; as well as for the U87MG cell line, used as a negative control, are depicted. * indicates statistical p value <0.05 using unpaired t-test with the Holm-Bonferroni correction for multiple comparisons. Tumors obtained from *in* *vivo* xenografts (1x10^6^ cells injection) in the brain striatum of BALB/c-Rag2^-/-^-IL2γc^-/-^ mice (n=6 per cell line) were detected using microPET and are shown circled by a dotted line (C). Tumor images corresponding to G63, G52 and G97C xenografts, as well as a negative control brain, are displayed, along with the corresponding quantification of maximum value of standardized caption (SUV_max_). A coronal section (200 mm thick) of a tumor originated by G97C was observed by means of a stereoscopic microscope (D) for tumor localization (black arrowhead) and a semi-thin section of the tumor was stained with hematoxylin-eosin (E).

In order to confirm the enrichment of GICs in the NS cultures, limiting dilution assays were performed (Figure 2A). NS cell lines required at least two-fold fewer cells to generate a secondary NS than the non GIC-enriched human GBM-derived cell line U87MG (11.4 cells for G63, 6.2 cells for G52, 5.4 cells for GN1C and 23.5 cells for U87MG). Likewise, while the U87MG cell line displayed 5.84±1.83% self-renewal capability in the clonogenic assay, the NS cell lines showed higher percentages of self-renewal: 57.39±15.93% for G63 (p=0.007); 61.11±21.68% for G52 (p=0.042) and 77.65±15.19% for GN1C (p=0.001) (Figure 2B). 

Besides self-renewal, an important feature of GICs is that they can generate tumors in immunodeficient mice. To test this ability, we performed intracranial injections of 1x10^6^ cells of G63, G97C and G52 into the striatum region of Rag2^-/-^IL2γc^-/-^ mice using a stereotactic frame. Four months after injection, all mice presented signs of disease and developed tumors that were detected by microPET, yielding SUV_max_ values of ~0.60 (Figure 2C). Upon necropsy, histopathological studies confirmed the presence of intracranial tumors resembling human GBM (Figure 2D-E). Together, these results indicate that the GBM-derived NS cell lines are enriched in GICs that show *in vitro* self-renewal and tumorigenic potential *in vivo*.

### miRNA and gene expression signatures of GICs change upon differentiation

**Figure 3:**
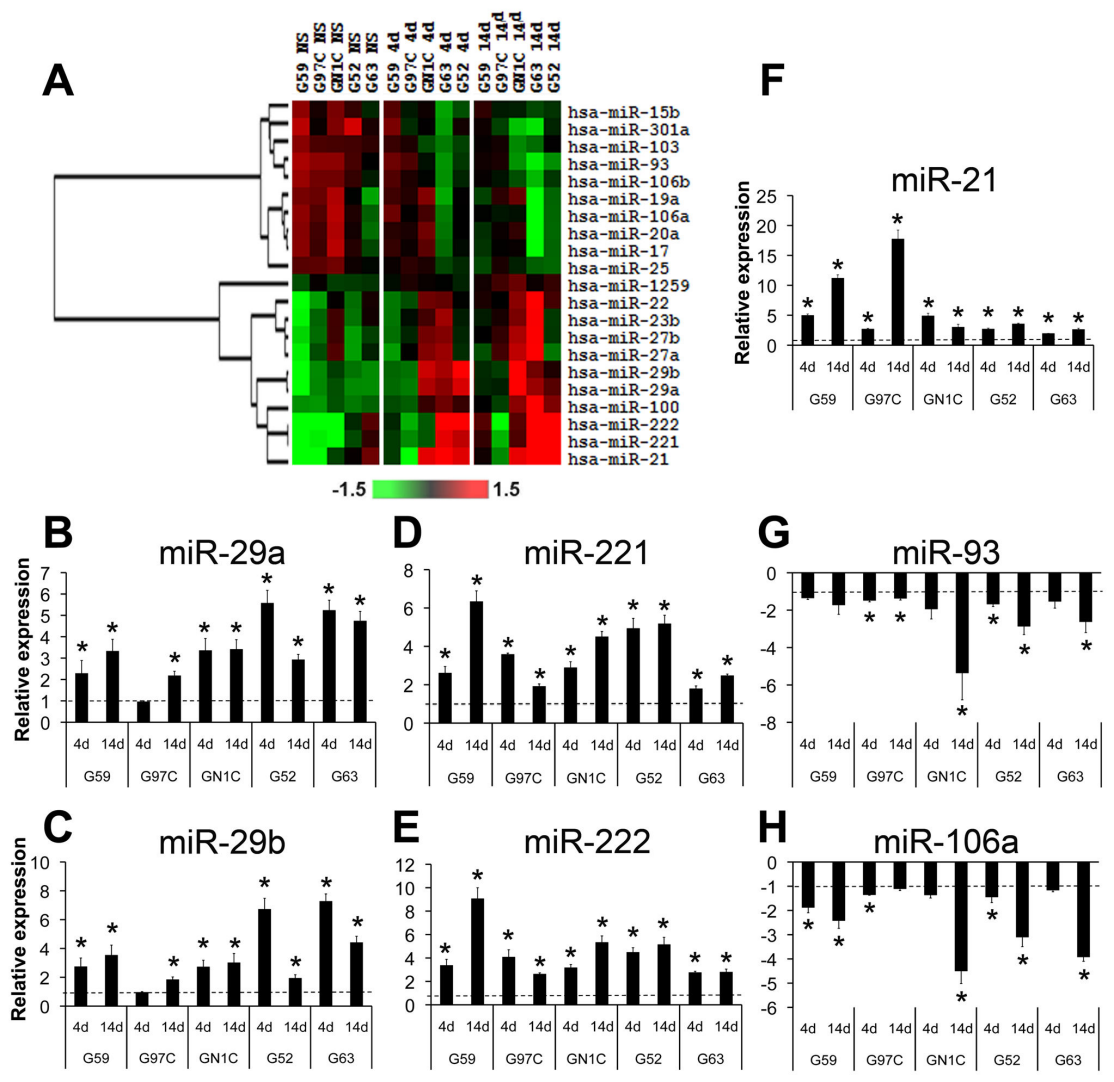
Microarray profiling of miRNA expression during NS differentiation. A heat map of a hierarchical clustering analysis of the microRNAs with differential expression between the NS cell lines at their basal state (NS) and after 4 (4d) and 14 (14d) days of induction of differentiation is displayed (A). Expression data are represented as log2Ratio and were mean centered for each miRNA. Validation of the differential expression of miR-29a (B), miR-29b (C), miR-221 (D), miR-222 (E), miR-21 (F), miR-93 (G) and miR-106a (H) was carried out by q-RT-PCR using specific TaqMan microRNA assays, normalizing their expression values with respect to RNU6B levels and to the NS state by calculating 2^-ΔΔCt^. * indicates statistical p value <0.05 using unpaired t-test with the Holm-Bonferroni correction for multiple comparisons; dotted lines indicate the basal expression level at the NS state.

We studied miRNA expression changes by microarray hybridization in the five NS cell lines before and after 4 and 14 days of differentiation. Data analysis yielded 21 miRNAs that consistently varied their expression during differentiation, including ten down-regulated and eleven up-regulated miRNAs (Figure 3A). Sixteen of these 21 deregulated miRNAs belong to genomic clusters, and 12 of them are accompanied by at least one miRNA of the same cluster displaying a similar expression pattern. In addition, eight of the down-regulated miRNAs belong to the three paralog clusters miR-17/92, miR-106a/363 and miR-106b/25, while three of the up-regulated miRNAs are part of the miR-23/24 paralog clusters. Expression changes were also validated by q-RT-PCR for seven miRNAs: 5 up-regulated (hsa-miR-21, hsa-miR-29a, hsa-miR-29b, hsa-miR-221 and hsa-miR-222) and 2 down-regulated (hsa-miR-93 and hsa-miR-106a) (Figure 3B-H). To begin to identify the putative target genes of these seven deregulated miRNAs during GIC differentiation, gene expression microarray experiments were performed in NS cell lines before and after differentiation. Data analysis defined the transcriptional signature of GIC differentiation, which included 932 probesets corresponding to 740 genes. Among them, 236 genes were potential targets of the seven deregulated miRNAs, according to TargetScan, PicTar, PITA, miRanda and microCosm predicted miRNA target databases (Table S2). Ingenuity Pathway Analysis (IPA) of these genes identified an enrichment in cellular functions including CNS processes, pluripotency and cancer, and in the Wnt/β-Catenin and Glutamate Receptor signaling pathways (Figure S2). These data indicate that the NS cell lines consistently show changes in the expression of coding and miRNA genes during the differentiation process. 

### Inhibition of miR-221/222 in differentiating GICs increased Nestin expression while decreased GFAP and TUJ1 levels

**Figure 4:**
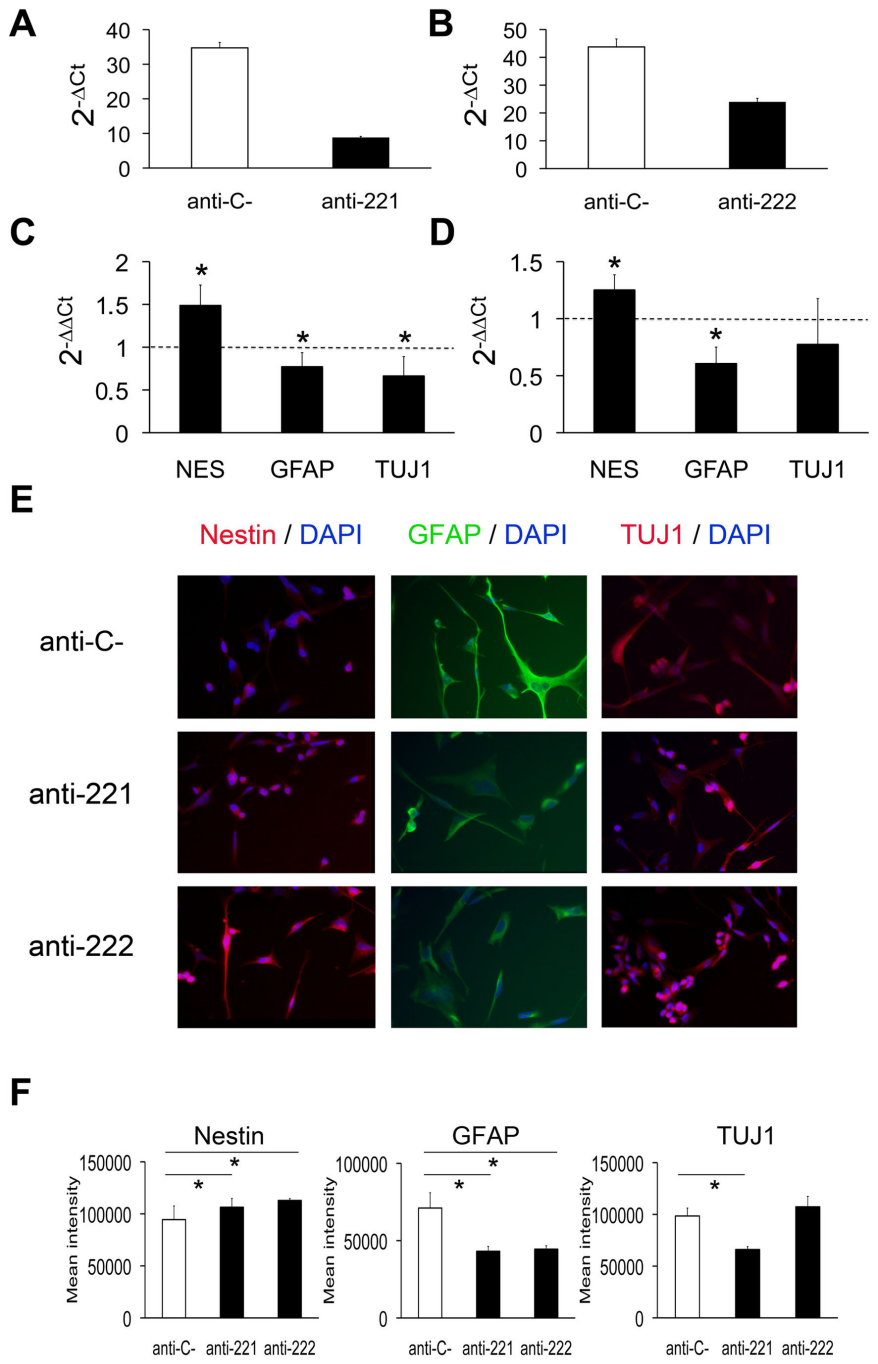
Inhibition of miR-221/222 in differentiating GICs increases Nestin expression and decreases GFAP and TUJ1 levels. miR-221 and miR-222 inhibition in GN1C cells growing in differentiation medium was carried out with specific anti-miRs and was confirmed by q-RT-PCR 14 days after transfection, compared to cells transfected with anti-miR negative control (anti-C-) (A, B). The expression of miR-221 and miR-222 was normalized with respect to RNU6B as 2^-ΔCt^. The expression of the progenitor marker Nestin (NES) as well as of astrocytic (GFAP) and neuronal (TUJ1) differentiation markers was also measured by q-RT-PCR and normalized with respect to GAPDH and to the cells transfected in parallel with the anti-miR negative control as 2^-ΔΔCt^ (C, D). Transfections were carried out in triplicate. Dotted lines indicate the expression level of GN1C cells transfected with anti-miR negative control. The corresponding protein expression levels of Nestin, GFAP and TUJ1 were visualized by immunofluorescence (E) using antibodies conjugated to FITC (green) or Texas Red (red) counterstained with DAPI (blue). Images were acquired at 20X magnification with a 739CCD camera coupled to an Axio Imager Z1 microscope (Carl Zeiss Inc.) using the Isis Imaging System software. Quantification of mean intensity for Nestin, GFAP and TUJ1 fluorescence is displayed (F). * indicates p value <0.05 using unpaired t-test or Mann-Whitney U test for statistical analysis and the Holm-Bonferroni correction for multiple comparisons.

miR-221/222 was the most significantly up-regulated miRNA cluster upon GIC differentiation (Figure 3A, D, E). In order to unveil the potential role of these miRNAs in GIC differentiation, we separately inhibited miR-221 and miR-222 in GN1C cells during 14 days of differentiation (Figure 4A-B). Analysis of mRNA expression of progenitor and differentiation markers resulted in an increase in Nestin levels for both miR-221 (1.48±0.23 fold, p=0.001; Figure 4C) and miR-222 inhibition (1.25±0.13 fold, p=0.005; Figure 4D), in comparison to control cells. In addition, a decrease in GFAP mRNA levels was observed after inhibiting either miR-221 (0.77±0.16 fold, p=0.012; Figure 4C) or miR-222 (0.60±0.14 fold, p<0.001; Figure 4D). Similar results were observed for TUJ1 expression, which was decreased after inhibition of miR-221 (0.66±0.22 fold, p=0.012; Figure 4C) and showed a tendency to decrease after miR-222 inhibition (0.77±0.40 fold, p=0.168; Figure 4D). The same expression profiles were confirmed in the G52 GIC line (Figure S3A-D). mRNA results were validated at the protein level by immunofluorescence (Figure 4E) and subsequent quantification (Figure 4F): Nestin expression increased in GN1C cells after inhibition of miR-221 (106383.4±8380.7 Mean Fluorescence Intensity (MFI), p=0,037) and miR-222 (112897.7±1819.4 MFI, p<0.001) compared to cells transfected with an anti-miR negative control (94505.4±13043.5 MFI), GFAP levels decreased in GN1C cells transfected with anti-miR-221 (43122.5±3114.4 MFI, p<0.001) and anti-miR-222 (44499.5±2202.2 MFI, p<0.001) compared to control cells (71093.5±9864.8 MFI), and TUJ1 levels were reduced upon miR-221 inhibition (66062.5±2774.7 vs. 98468.3±7645.5 MFI, p<0.001) but not after inhibiting miR-222 (Figure 4E-F). In summary, inhibition of the miRNAs of the cluster miR-221/222 in differentiating GICs decreases the expression of astrocytic and neuronal lineage markers, which suggests their involvement in GIC-induced differentiation.

### miR-21 over-expression in GICs induces GFAP expression, decreases Nestin levels and targets *SPRY1*

**Figure 5:**
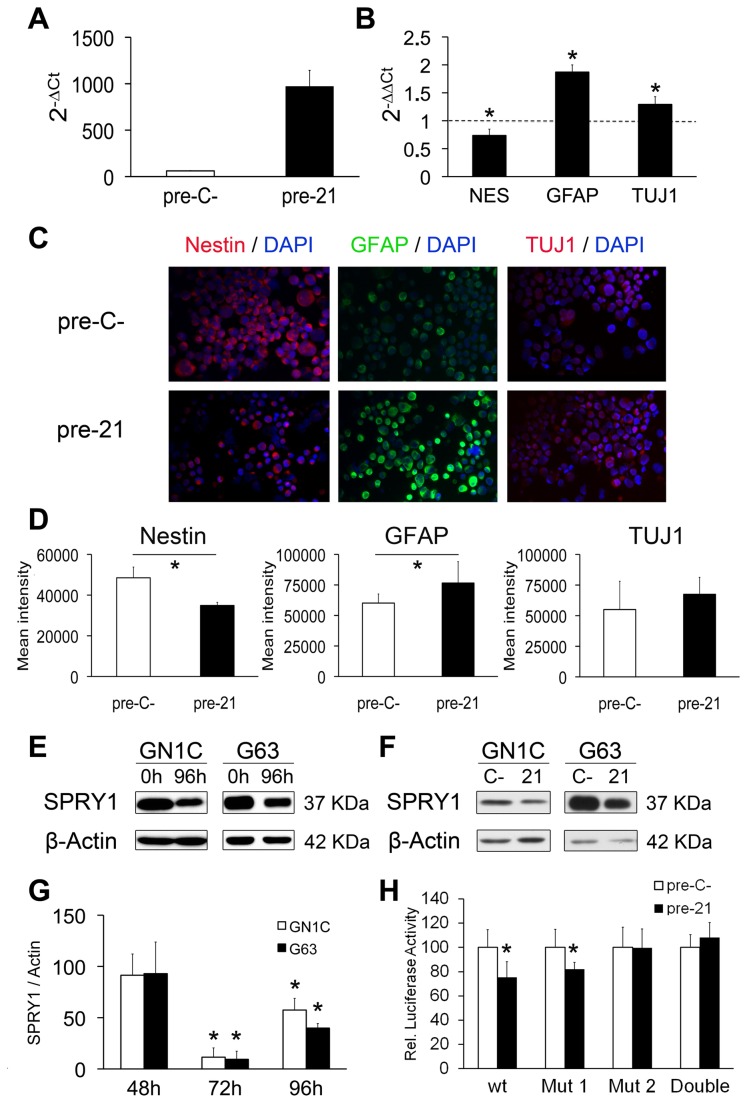
miR-21 over-expression in GICs induces GFAP expression, decreases Nestin levels and targets *SPRY1*. miR-21 over-expression was analyzed by q-RT-PCR 7 days after transfection and growth in NS medium (A). 2^-ΔCt^ was calculated as miR-21 expression relative to RNU6B expression for GN1C cells transfected with pre-miR negative control (pre-C-) or miR-21 precursor (pre-21). mRNA expression levels of Nestin (NES) as well as astrocytic (GFAP) and neuronal (TUJ1) differentiation markers were measured by q-RT-PCR (B). 2^-ΔΔCt^ was calculated relative to GAPDH expression and GN1C cells transfected with pre-miR negative control (dotted lines). Transfections were carried out in triplicate. Protein expression levels of Nestin, GFAP and TUJ1 were visualized by immunofluorescence (C), 7 days after transfection of GN1C cells with miR-21 precursor (pre-21) or pre-miR negative control (pre-C-). Images were acquired at 20X magnification with a 739CCD camera coupled to an Axio Imager Z1 microscope (Carl Zeiss Inc.) using the Isis Imaging System software. Quantification of mean intensity for Nestin, GFAP and TUJ1 fluorescence is displayed (D). SPRY1 and β-Actin (loading control) protein levels were measured by Western blot in the GN1C and G63 cell lines at the NS state (0h) and after 96 hours (96h) of induction of their differentiation (E), as well as 96 hours after transfection with pre-miR-21 (21) or a pre-miR negative control (C-) (F). A time-course study of SPRY protein expression by Western blot was also performed at 48, 72 and 96 hours after transfection with pre-miR-21 (21) or a pre-miR negative control (C-) (G). Quantification of at least three independent replicates was performed using ImageJ software and is shown as SPRY1/β-Actin expression relative to pre-miR negative control transfected cells (100%). Specific binding of miR-21 to the 3´-UTR binding sites of *SPRY1* was assessed in the GN1C cell line using luciferase assays and site-directed mutagenesis (H) “Mut 1” corresponds to mutation of the site in position chr4:124324121-124324128 and “Mut 2” to chr4:124323893-124323900. Transfections were carried out in triplicate. * indicates p value <0.05 in unpaired t test statistical analysis using the Holm-Bonferroni correction for multiple comparisons.

miR-21 was the most up-regulated miRNA during GIC differentiation (Figure 3A, F). To functionally test this finding, we over-expressed miR-21 in GN1C cells at the NS state for seven days (Figure 5A). miR-21 ectopic expression induced an increase in GFAP (1.87±0.12 fold, p<0.001) and TUJ1 (1.29±0.13 fold, p=0.001) mRNA expression levels, and a decrease of Nestin expression (0.73±0.11 fold, p<0.001), compared to NS transfected with the pre-miR negative control (Figure 5B). Similar results were obtained in the G63 GIC line (Figure S3E-F). Expression changes were confirmed at the protein level by immunofluorescence (Figure 5C-D): Nestin staining was markedly reduced in GN1C cells over-expressing miR-21 in comparison to control cells (34902±1559.9 vs. 48508.8±5285.26 MFI, p=<0.001), while GFAP immunostaining was increased (76563.8±17485.6 vs. 60142.1±7436 MFI, p=0.02). TUJ1 expression showed a tendency to increase upon miR-21 over-expression (67460.9±13871.2 *vs.* 55015±23088.4 MFI, p=0.26). These results point to the involvement of miR21 in GIC differentiation. Triple immunofluorescence staining showed that miR-21 over-expression in GICs induced the co-expression of both GFAP and TUJ1 in the transfected cells (Figure S1B), such as was observed in GICs cultured in differentiation medium (Figure S1A).

One of the miR-21 predicted targets showing down-regulated expression during GIC differentiation was *SPRY1* (Sprouty1) (Figure S4A), a gene that inhibits neural differentiation in mouse embryonic stem cells [29] and has been previously reported as miR-21 target in fibroblasts [30] and keratinocytes [31]. Western blot analysis showed a marked decrease of SPRY1 after 4 days of differentiation in GN1C and G63 cell lines (Figure 5E). To assess whether this down-regulation was caused by miR-21 up-regulation, transfection experiments with a miR-21 precursor were carried out. Results showed that miR-21 over-expression at the NS state induced a similar decrease in SPRY1 protein expression in both cell lines with maximum inhibition after 72 hours (88.55% reduction, p<0.001 for GN1C; 90.62%, p=0.004 for G63) (Figure 5F-G). Accordingly, we identified two potential binding sites for miR-21 in the 3´-UTR of *SPRY1* (Figure S4B). Subsequent dual luciferase assays demonstrated regulation of the 3´-UTR of *SPRY1* by miR-21, resulting in a significant decrease (24.74%, p=0.002) of the reporter luciferase activity that was abolished by the mutation of the more distal putative binding site for this miRNA (Figure 5H). These data show that miR-21 targets *SPRY1* by direct 3´-UTR binding, suggesting that miR-21 is probably involved in the decrease of SPRY1 protein expression observed during GIC differentiation.

### Over-expression of miR-29a/29b promotes apoptosis of GICs by inhibiting MCL1 protein expression

**Figure 6:**
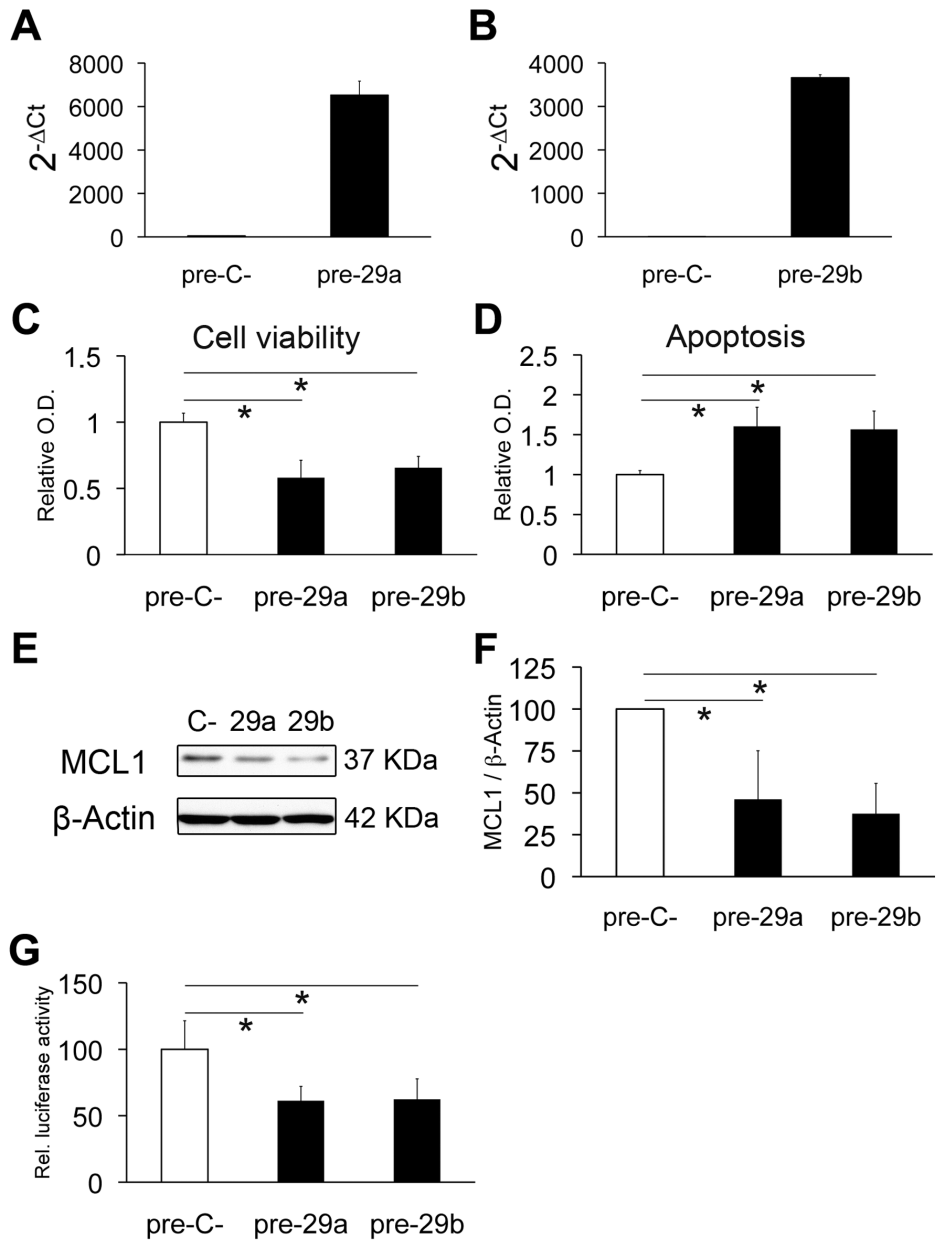
Over-expression of miR-29a/29b targets *MCL1* and promotes apoptosis in GICs. miR-29a/b over-expression in GN1C cells transfected with pre-miR-29a (pre-29a) or pre-miR-29b (pre-29b) compared to pre-miR negative control (pre-C-) was confirmed by q-RT-PCR 4 days after transfection (A, B). Cell viability assays using MTS (C) and apoptosis assessment by Cell Death Detection kit (D) were carried out 4 days after transfection. MCL1 protein levels were also measured by Western blot at the same time point, using β-Actin as loading control (E). Quantification of Western blots was performed with ImageJ (F) and is displayed as the MCL1/β-actin ratio relative to the negative control (100%). Regulation of the 3´-UTR of *MCL1* by miR-29a and miR-29b was analyzed by luciferase assays (G). All experiments were carried out at least in triplicate. * indicates p value <0.05 in unpaired t test or Mann-Whitney U test, using the Holm-Bonferroni correction for multiple comparisons.

The expression of each of the miRNAs of the miR-29a/29b cluster showed a 3- to 4-fold increase in average during GIC differentiation (Figure 3A, B, C). However, over-expression of miR-29a and miR-29b in GN1C cells at the NS state (Figure 6A-B) did not induce changes in the expression of differentiation markers, but a significant decrease in cell viability and increased apoptosis were observed (Figure 6C-D). Previous studies have demonstrated that miR-29a/29b can promote apoptosis by targeting *MCL1* mRNA in cholangiocarcinoma cell lines [32]. Accordingly, Western blot assays showed a reduction in MCL1 protein expression upon over-expression of miR-29a (53.87% decrease, p<0.001) and miR-29b (62.49% decrease, p<0.001) (Figure 6E-F), resulting in 1.6±0.24 fold (p=0.0002) and 1.56±0.23 fold (p=0.0001) increased apoptosis, respectively (Figure 6D). All these results were validated in the G63 GIC line (Figure S5). Furthermore, dual luciferase reporter experiments demonstrated targeting of the 3´-UTR of *MCL1* by miR-29a and miR-29b, resulting in a significant decrease (38.78%, p<0.001 for miR-29a; 37.67%, p=0.003 for miR-29b) of luciferase activity (Figure 6G). In conclusion, miR-29a and miR-29b are able to target the anti-apoptotic protein MCL1 and promote apoptosis in GICs.

## Discussion

Our work provides for the first time a high-throughput analysis of miRNA expression during differentiation of GICs [9]. This model resembles the clinical conditions in which the therapeutic induction of differentiation of GICs should be achieved more accurately than the ones based on growth factor withdrawal, since the tumor microenvironment is rich in growth factors [33]. Probably due to this methodological difference, we did not confirm previous findings that reported a pro-differentiation role for miR-124 and miR-137 on human GICs [14]. On the contrary, we found that in our miRNA microarray data these miRNAs were expressed at very low levels (miR-124) or none at all (miR-137). Moreover, Silber et al. also found that the expression of miR-124 and miR-137 was associated with neuronal-like opposed to astrocyte-like differentiation, while the most up-regulated miRNAs in our study (miR-21, miR-29a, miR-29b, miR-221 and miR-222) are associated to the GBM subclass showing a miRNA expression profile evocative to that of astrocytic precursors, according to the five subgroups of GBM defined by Kim et al. based on miRNA expression profiles [34]. Nevertheless, triple immunofluorescence staining revealed that our differentiation method induced the expression of both astrocytic and neuronal markers in the differentiating GICs simultaneously. These results might suggest a differentiation of these cells towards the neuronal lineage, but retaining the expression of GFAP that is usually restricted to neural precursors in the neuronal lineage [35], while it is abundantly expressed in the astrocytic lineage. This aberrant marker expression in differentiating GICs has been previously reported by other groups [36].

Analogously to what has been reported for the differentiation of normal neurons [37], most of the miRNAs that changed their expression levels upon GIC differentiation in our model belong to the same miRNA clusters, and several paralog clusters are involved. For instance, the paralog miRNA clusters miR-106a/363 (integrated by miR-106a, miR-363, miR-92-2, miR-19b-2, miR-20 and miR-18b), miR-106b/25 (compound of miR-106b, miR-25 and miR-93) and miR-17/92 (comprising miR-17, miR-18a, miR-19a, miR-20a, miR-19b-1 and miR-92a-1) are down-regulated upon differentiation, while clusters miR-29a/29b and miR221/222 are strongly up-regulated, suggesting an important role for coordinate regulatory miRNA networks during GIC differentiation. To assess the significance of these two up-regulated miRNA clusters in the differentiation process, we performed transfection experiments using precursors or inhibitors of these miRNAs and analyzed the expression of differentiation markers. Cluster miR-29a/29b did not induce the expression of the studied differentiation markers, but sensitized the cells to apoptosis by targeting MCL1, a *bona-fide* target of the miR-29 family [32]. Interestingly, MCL1 is the most over-expressed protein of the BCL2 family in the majority of malignant gliomas, and neutralization of MCL1 in glioma cells has been reported to induce apoptosis and increase chemotherapy-induced apoptosis [38], suggesting that miR-29a/29b over-expression could be studied as a possible therapy for GBM. The up-regulation of cluster miR-221/222 that we observed upon GIC differentiation is more controversial, since this cluster has been found over-expressed in GBM compared to non-transformed tissue [39], being particularly associated to the astrocytic GBM subclass [34]. Conversely, both miRNAs have been shown to inhibit proliferation in the TF-1 erythroleukemic cell line and to reduce the stem cell repopulating activity of cord blood CD34+ cells through inhibition of KIT [40]. Of note, KIT amplification is a frequent alteration in GBM [41]. Thus, these miRNAs probably can exert pro-oncogenic or tumor suppressor functions depending on the cellular context. Regarding neural cell differentiation, miR-221 has been found highly up-regulated upon nerve growth factor-induced neuronal-like differentiation of PC12 rat pheochromocytoma cells [42]. miR-221 could be exerting a similar role during GIC differentiation.

One of the most surprising findings of this work is the pro-differentiation role of miR-21 over-expression in GICs. miR-21 is regarded as an onco-miR in GBM, as well as in other tumors, and its over-expression has been associated to poor clinical outcome [43]. Indeed, miR-21 has shown a widespread involvement in the inhibition of tumor suppressor genes in GBM cells, targeting multiple components of the p53, transforming growth factor-β (TGF-β) and mitochondrial apoptosis pathways [44]. Consequently, the inhibition of miR-21 expression with therapeutic intent has been suggested as a possible treatment for GBM and some preliminary *in vitro* and *in vivo* assays have provided promising results. For instance, it has been reported that the inhibition of miR-21 in GBM cells as well as in glioma xenotransplant-bearing mice promotes apoptotic cell death of the tumor cells [45,46], but no studies of its effects on survival were performed, as all animals were sacrificed 6 days after treatment with LNA-anti-miR-21. In this regard, our finding of the involvement of miR-21 in the positive regulation of differentiation of GICs suggests that miR-21 inhibition could increase the stemness of this population, which is considered the probable source of tumor relapses after GBM treatment. Of note, analogous data have been published by other groups concerning the role of miR-21 in the biology of stem cells. For example, the neural repressor REST maintains the self-renewal capacity and the pluripotency of murine embryonic stem cells by suppressing miR-21, suggesting that miR-21 could behave as an anti-proliferative factor in these cells by targeting Nanog and Sox2 [47]. These data support a pro-differentiation role of miR-21 in the stem cell context. Furthermore, in this study we demonstrate that *SPRY1* is a direct target of miR-21 in GICs, and it has been reported that *SPRY1* inhibition promotes neural differentiation in mouse embryonic stem cells [29]. SPRY1, along with SPRY2, has a role as negative feedback regulator of FGF signaling also in the mouse ventricular zone of the brain, where it regulates cortical proliferation, differentiation and the expression of genes that modulate progenitor identity [48]. Taking these data into account, we suggest that miR-21 could induce the differentiation of GICs by targeting *SPRY1* in these cells. Further studies are warranted to elucidate the effects of miR-21 inhibition on GICs and GBM tumor relapses in an *in vivo* setting, which would be particularly interesting in the context of evaluating miR-21 inhibition as a possible treatment for GBM.

In conclusion, we have found that some miRNAs with oncogenic roles in GBM, such as miR-21 and the miR-221/222 cluster, are positive regulators of GIC differentiation in presence of growth factors. These findings suggest that their inhibition with the intent to improve GBM treatment might not have the desired effects on avoiding relapses, what should be taken into account for future studies. On the contrary, the miR-29a/29b cluster could promote GIC apoptosis and also improve bulk tumor killing in GBM by targeting MCL1, being a promising candidate to design future treatments for GBM aimed to avoid recurrence.

## Supporting Information

Figure S1 **Triple immunofluorescence staining for Nestin, GFAP and TUJ1 in GN1C GICS upon differentiation or miR-21 over-expression.** GN1C cells were cultured during 14 days in NS or differentiation media (14d) (**A**) or for 7 days in NS medium after pre-miR-21 (pre-21) or pre-miR negative control 1 (pre-C-) over-expression (**B**), and Nestin (Alexa fluor 488), GFAP (Alexa fluor 568) and TUJ1 (Alexa fluor 647) were detected by immunofluorescence. Images were acquired with a Leica SP5-II confocal microscope using a 20x/0.7 NA water immersion objective.
(TIF)

Figure S2 **Putative targets of the 7 miRNAs with significant changes of expression during GIC differentiation are involved in cell functions and canonical pathways relevant to neural processes, pluripotency and cancer.** Among the 740 genes with differential expression upon GIC differentiation, public databases for prediction of miRNA targets identified 236 putative targets of the 7 miRNAs that significantly changed their expression during that process. IPA functional analysis of these putative targets identified their involvement in functions (**A**) and canonical pathways (**B**) related to neural processes, cancer and pluripotency (underlined). Only the 10 most significant pathways or functions among the ones with associated p value below 0.05 (corresponding with a threshold of 1.30 in the axis representing –log(p value), indicated by a yellow line in the graphs) are depicted. The Wnt/β-Catenin pathway included many of the putative miRNA targets altered during differentiation (**C**), most of them down-regulated (green), and an important inhibitor of this pathway, *DKK1*, was found up-regulated (red) during differentiation.
(TIF)

Figure S3 **Validation of the functional studies of miR-221/221 inhibition and miR-21 over-expression in additional GIC lines.** G52 cells transfected with anti-miR-221 (anti-221), anti-mir-222 (anti-222) and anti-miR negative control 1 (anti-C-) were cultured for 14 days in differentiation medium (**A**-**D**). miR-21 (pre-21) or pre-miR negative control 1 (pre-C-) transfected G63 cells were cultured in NS medium during 7 days (**E**-**F**). Cells were assayed for the expression of miR-221 (**B**), miR-222 (**D**) or miR-21 (**F**) by q-RT-PCR. 2^-ΔCt^ was calculated as miRNA expression relative to RNU6B expression. mRNA expression levels of Nestin (NES) as well as astrocytic (GFAP) and neuronal (TUJ1) differentiation markers were measured by q-RT-PCR (**A**, **C**, **E**). 2^-ΔΔCt^ was calculated relative to GAPDH expression and to GICs transfected with the corresponding negative control (dotted lines). At least two independent transfections were performed. *, statistical p value <0.05 using unpaired t test and Holm-Bonferroni correction.
(TIF)

Figure S4 ***SPRY1*, down-regulated upon GIC differentiation, displays two putative binding sites for miR-21 in its 3´-UTR.** *SPRY1* (underlined) is one of the 10 most down-regulated genes among the putative targets of our 7 selected miRNAs with differential expression upon GIC differentiation, as shown in the Top Molecules display of the IPA analysis (**A**). Scrutiny with the PITA prediction algorithm (**B**) identified a seed perfect match putative binding site for miR-21 at position chr4:124324121-124324128 (Genome Browser hg19 assembly) (black font highlighted in green), and another more degraded possible site at position chr4:124323893-124323900 (light green font). M means mismatch and W wobble pair in the pairing between the seed of miR-21 (SEED) and the putative binding site. The annealing positions of the primers used for amplification and subsequent cloning of the 3´-UTR are underlined.
(TIF)

Figure S5 **Over-expression of the miRNAs of the miR-29a/29b cluster in G63** **cells at the NS state induces apoptosis and inhibits MCL1**. miR-29a/b over-expression in G63 cells transfected with pre-miR-29a (pre-29a) or pre-miR-29b (pre-29b) compared to pre-miR negative control (pre-C-) was confirmed by q-RT-PCR 4 days after transfection (A, B). Cell viability assays using MTS (C) and apoptosis assessment by Cell Death Detection kit (D) were carried out 4 days after transfection. MCL1 protein levels were assessed by Western blot two days after transfection, using β-Actin as loading control (E). Quantification of Western blots was performed with ImageJ (F) and is displayed as the MCL1/β-Actin ratio relative to the negative control (100%). At least two independent transfections were carried out. *, p value <0.05 in unpaired t test or Mann-Whitney U test, using the Holm-Bonferroni correction for multiple comparisons.
(TIF)

Table S1 **Primers for 3´-UTR cloning and site directed mutagenesis.**
(DOCX)

Table S2 **Excel file containing the expression data of the 932 genes differentially expressed upon GIC differentiation as normalized logValues, indicating the genes that are also putative targets of any of our 7 validated miRNAs (Gene expression worksheet) and a summary of the results from the analysis of miRNA target prediction databases (miRNA target prediction worksheet).**
(XLSX)
