# Supplementary figures and images for: Involvement of miRNAs in the Differentiation of Human Glioblastoma Multiforme Stem-Like Cells
Source: PLoS One. 2013 Oct 14;8(10):e77098. doi: 10.1371/journal.pone.0077098 (PMC3796557; doi:10.1371/journal.pone.0077098)

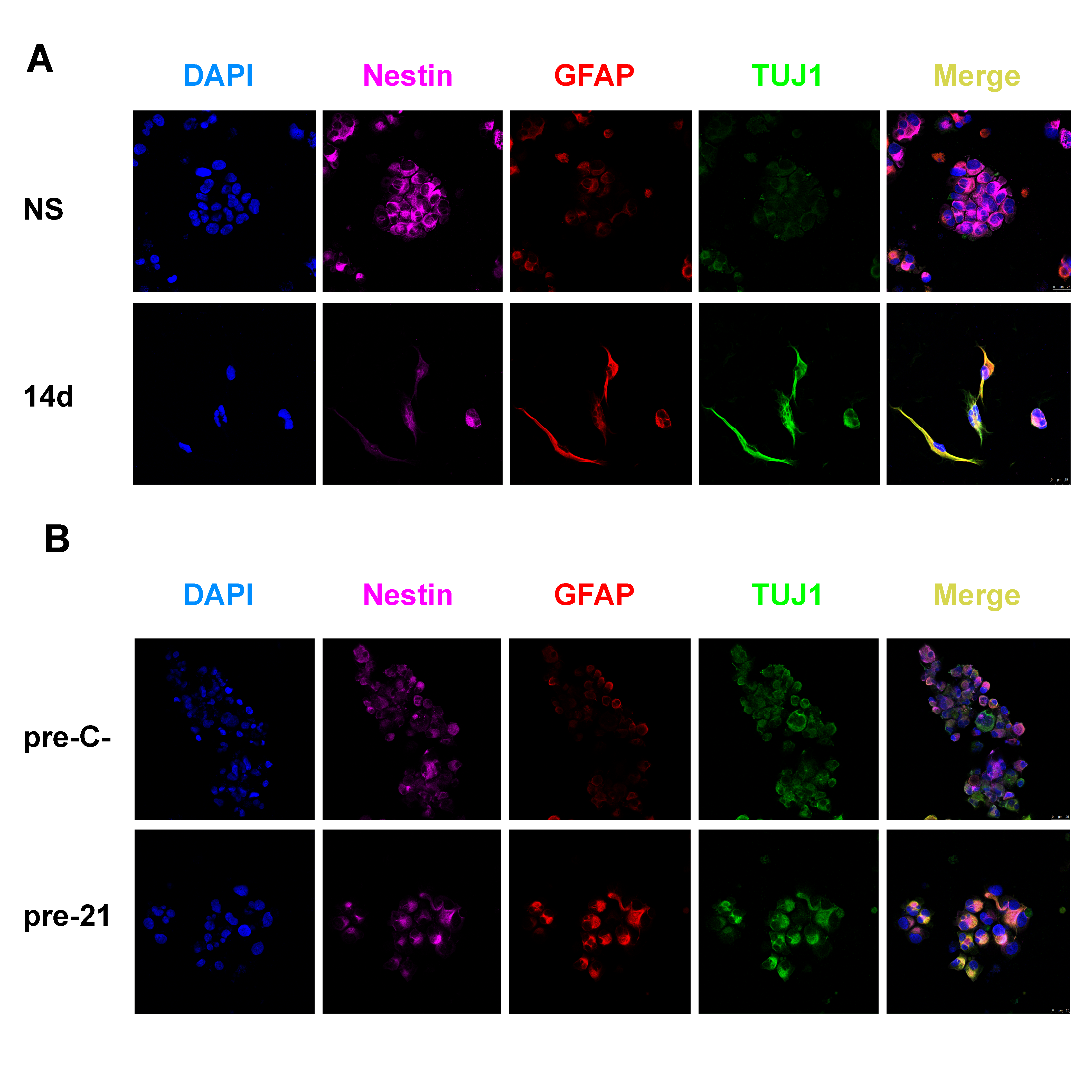

Supplement: Figure S1 — Triple immunofluorescence staining for Nestin, GFAP and TUJ1 in GN1C GICS upon differentiation or miR-21 over-expression. GN1C cells were cultured during 14 days in NS or differentiation media (14d) (A) or for 7 days in NS medium after pre-miR-21 (pre-21) or pre-miR negative control 1 (pre-C-) over-expression (B), and Nestin (Alexa fluor 488), GFAP (Alexa fluor 568) and TUJ1 (Alexa fluor 647) were detected by immunofluorescence. Images were acquired with a Leica SP5-II confocal microscope using a 20x/0.7 NA water immersion objective. (TIF) [file pone.0077098.s001.tif]

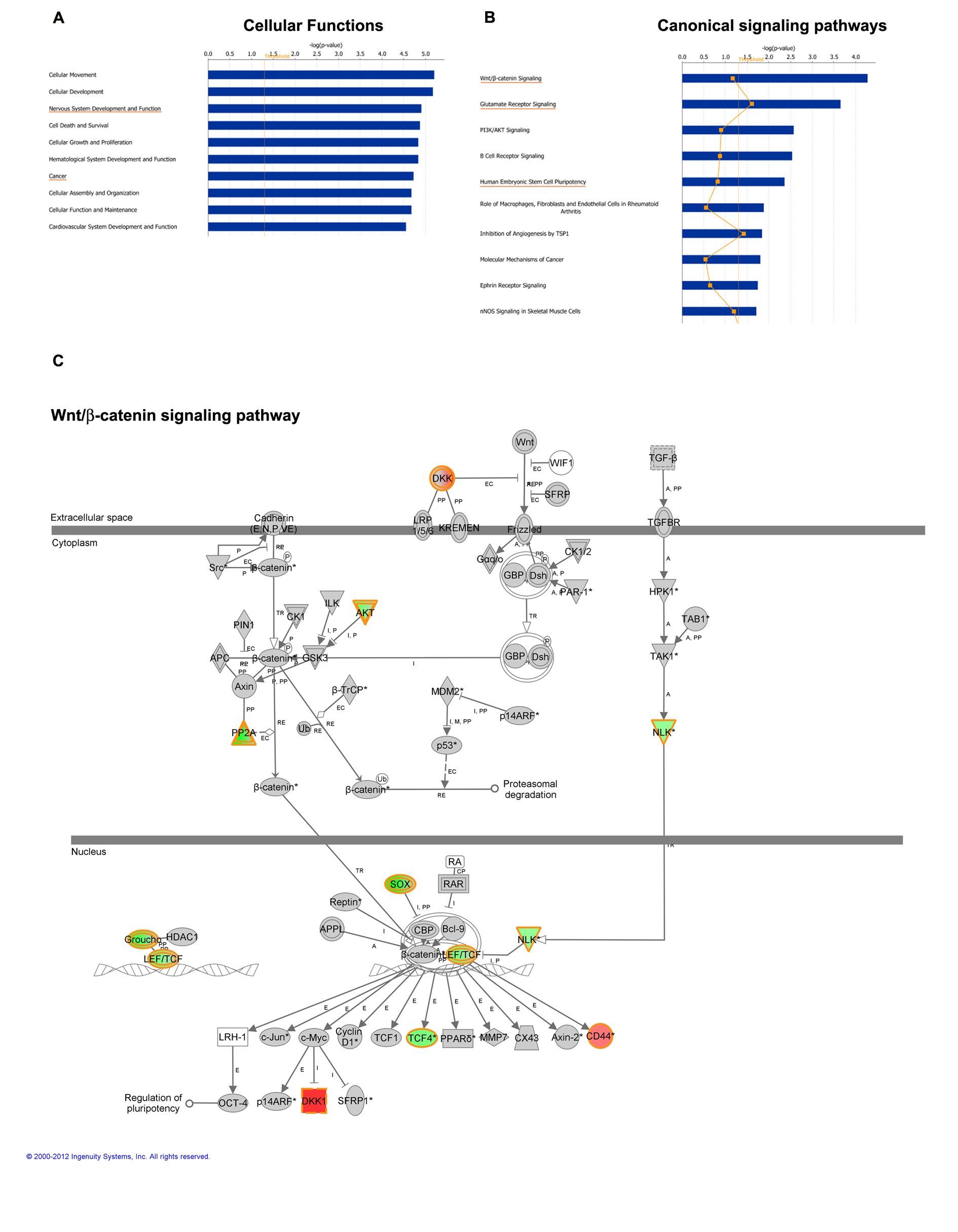

Supplement: Figure S2 — Putative targets of the 7 miRNAs with significant changes of expression during GIC differentiation are involved in cell functions and canonical pathways relevant to neural processes, pluripotency and cancer. Among the 740 genes with differential expression upon GIC differentiation, public databases for prediction of miRNA targets identified 236 putative targets of the 7 miRNAs that significantly changed their expression during that process. IPA functional analysis of these putative targets identified their involvement in functions (A) and canonical pathways (B) related to neural processes, cancer and pluripotency (underlined). Only the 10 most significant pathways or functions among the ones with associated p value below 0.05 (corresponding with a threshold of 1.30 in the axis representing –log(p value), indicated by a yellow line in the graphs) are depicted. The Wnt/β-Catenin pathway included many of the putative miRNA targets altered during differentiation (C), most of them down-regulated (green), and an important inhibitor of this pathway, DKK1, was found up-regulated (red) during differentiation. (TIF) [file pone.0077098.s002.tif]

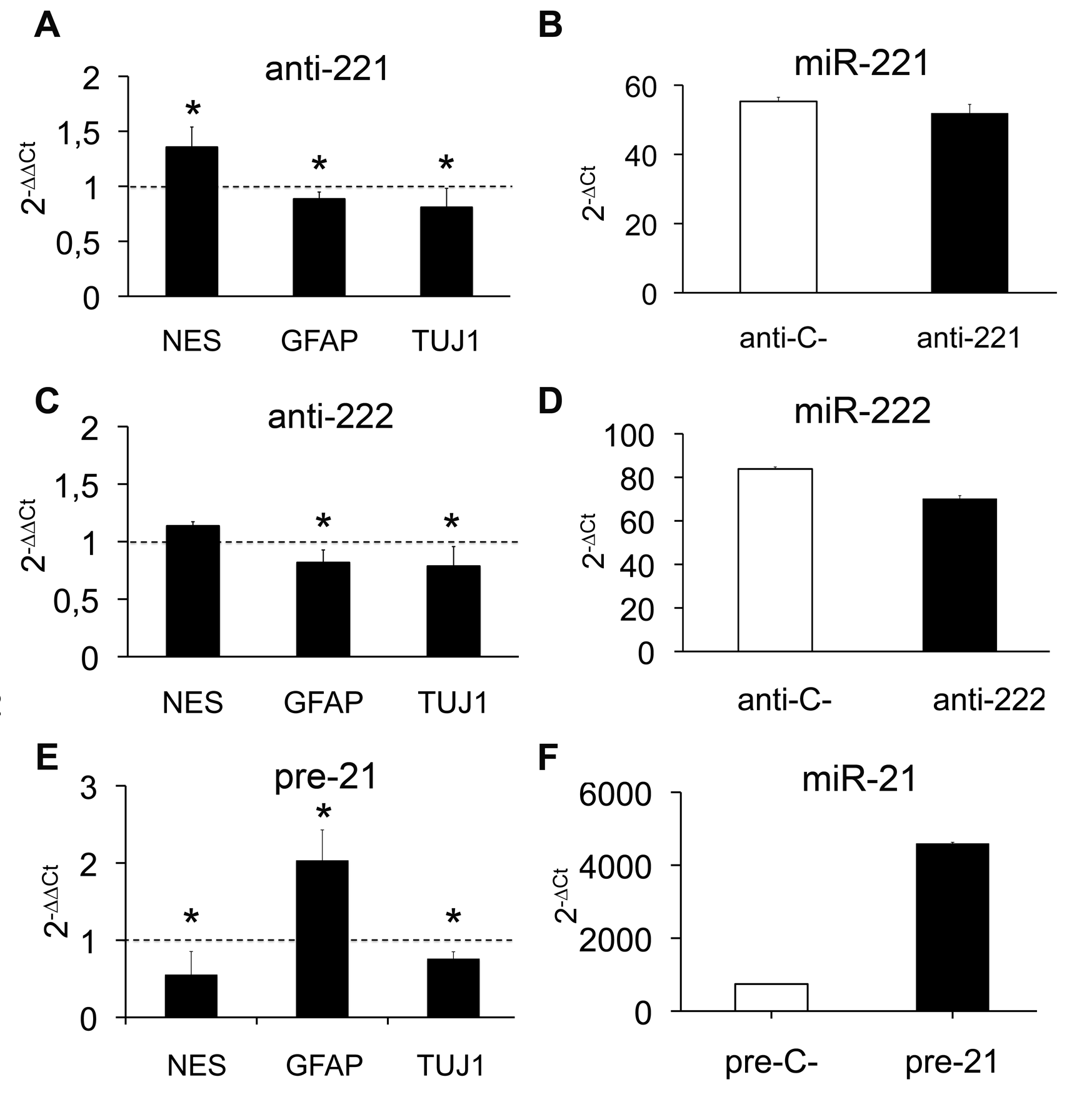

Supplement: Figure S3 — Validation of the functional studies of miR-221/221 inhibition and miR-21 over-expression in additional GIC lines. G52 cells transfected with anti-miR-221 (anti-221), anti-mir-222 (anti-222) and anti-miR negative control 1 (anti-C-) were cultured for 14 days in differentiation medium (A-D). miR-21 (pre-21) or pre-miR negative control 1 (pre-C-) transfected G63 cells were cultured in NS medium during 7 days (E-F). Cells were assayed for the expression of miR-221 (B), miR-222 (D) or miR-21 (F) by q-RT-PCR. 2-ΔCt was calculated as miRNA expression relative to RNU6B expression. mRNA expression levels of Nestin (NES) as well as astrocytic (GFAP) and neuronal (TUJ1) differentiation markers were measured by q-RT-PCR (A, C, E). 2-ΔΔCt was calculated relative to GAPDH expression and to GICs transfected with the corresponding negative control (dotted lines). At least two independent transfections were performed. *, statistical p value <0.05 using unpaired t test and Holm-Bonferroni correction. (TIF) [file pone.0077098.s003.tif]

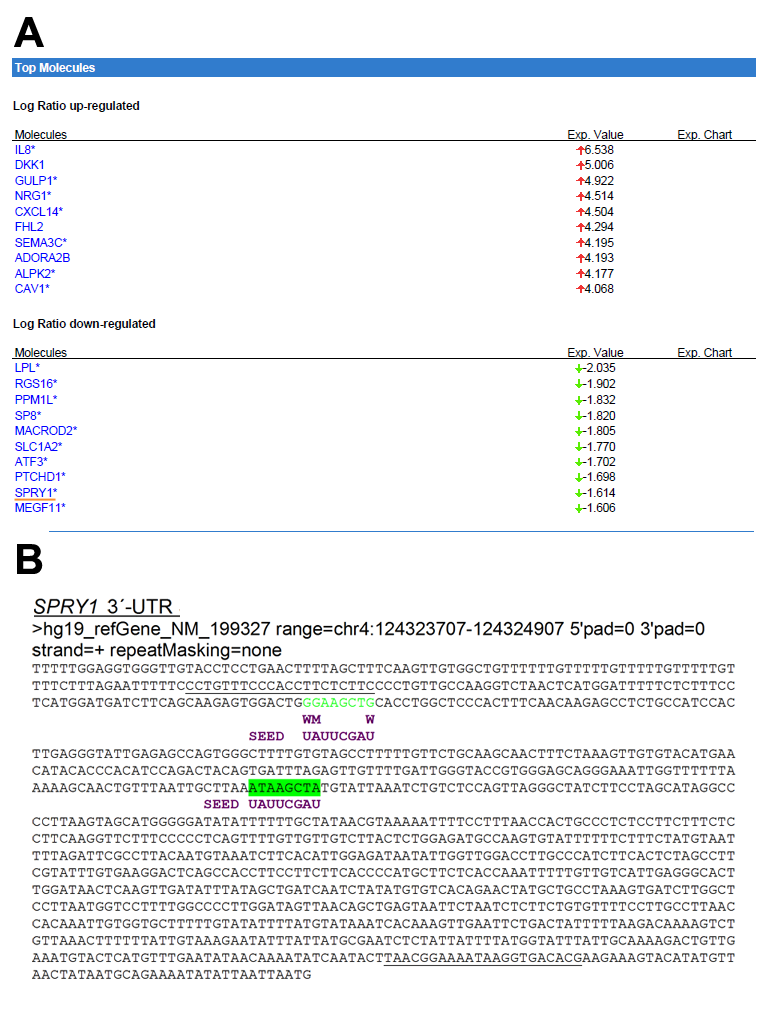

Supplement: Figure S4 — SPRY1, down-regulated upon GIC differentiation, displays two putative binding sites for miR-21 in its 3´-UTR. SPRY1 (underlined) is one of the 10 most down-regulated genes among the putative targets of our 7 selected miRNAs with differential expression upon GIC differentiation, as shown in the Top Molecules display of the IPA analysis (A). Scrutiny with the PITA prediction algorithm (B) identified a seed perfect match putative binding site for miR-21 at position chr4:124324121-124324128 (Genome Browser hg19 assembly) (black font highlighted in green), and another more degraded possible site at position chr4:124323893-124323900 (light green font). M means mismatch and W wobble pair in the pairing between the seed of miR-21 (SEED) and the putative binding site. The annealing positions of the primers used for amplification and subsequent cloning of the 3´-UTR are underlined. (TIF) [file pone.0077098.s004.tif]

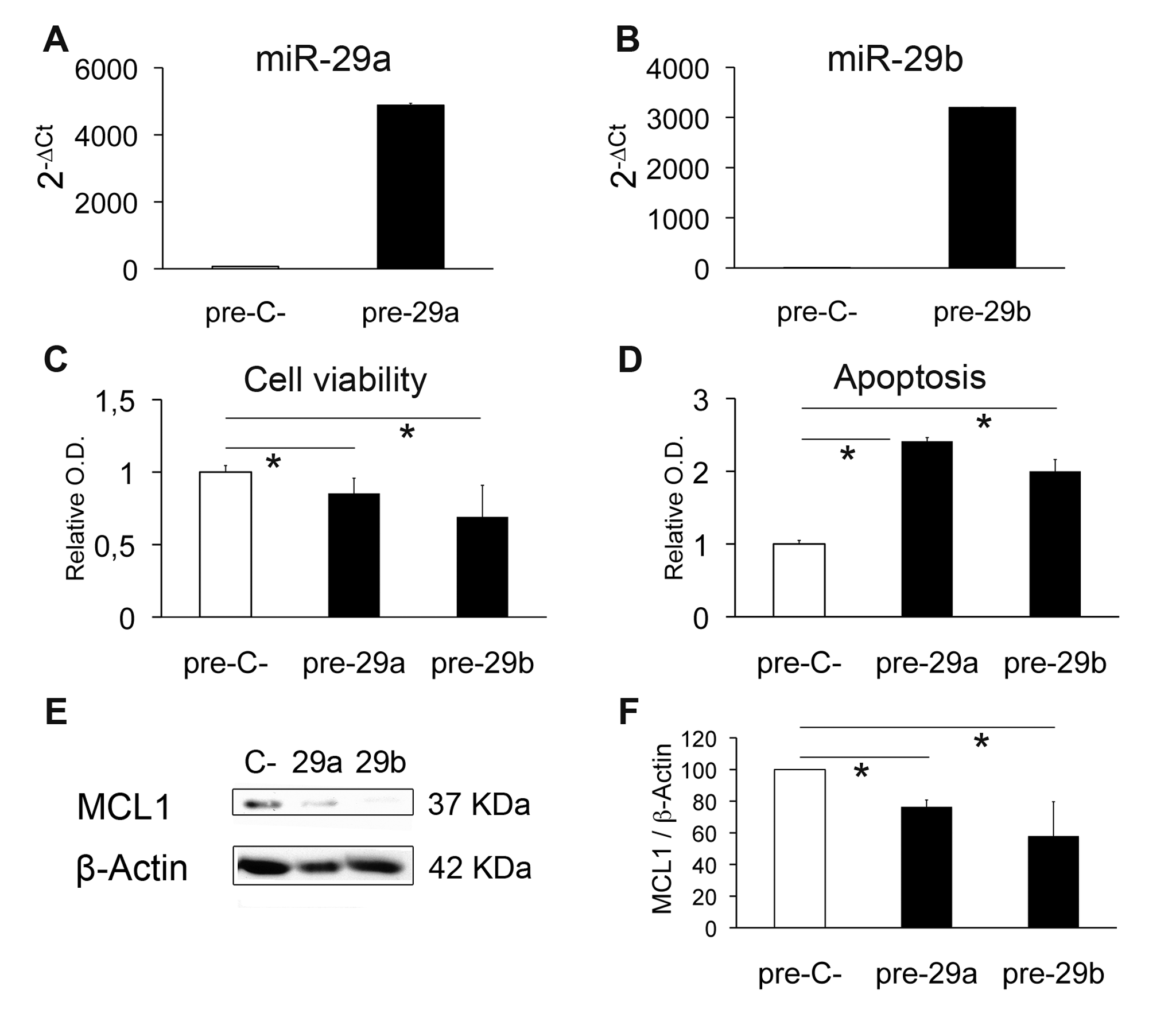

Supplement: Figure S5 — Over-expression of the miRNAs of the miR-29a/29b cluster in G63 cells at the NS state induces apoptosis and inhibits MCL1. miR-29a/b over-expression in G63 cells transfected with pre-miR-29a (pre-29a) or pre-miR-29b (pre-29b) compared to pre-miR negative control (pre-C-) was confirmed by q-RT-PCR 4 days after transfection (A, B). Cell viability assays using MTS (C) and apoptosis assessment by Cell Death Detection kit (D) were carried out 4 days after transfection. MCL1 protein levels were assessed by Western blot two days after transfection, using β-Actin as loading control (E). Quantification of Western blots was performed with ImageJ (F) and is displayed as the MCL1/β-Actin ratio relative to the negative control (100%). At least two independent transfections were carried out. *, p value <0.05 in unpaired t test or Mann-Whitney U test, using the Holm-Bonferroni correction for multiple comparisons. (TIF) [file pone.0077098.s005.tif]
