# Supplementary material for: Involvement of miRNAs in the Differentiation of Human Glioblastoma Multiforme Stem-Like Cells
Source: PLoS One. 2013 Oct 14;8(10):e77098. doi: 10.1371/journal.pone.0077098 (PMC3796557; doi:10.1371/journal.pone.0077098)
Supplement: Table S1 — Primers for 3´-UTR cloning and site directed mutagenesis. (DOCX) [file pone.0077098.s006.docx]

# Supplemental Table S1

**Table S1. Primers for 3´-UTR cloning and site directed mutagenesis**

| Name | | Sequence (5´-3´) | Purpose |
| --- | --- | --- | --- |
| MCL1-XFW | GGGAAACTCGAGCATTTGAGTTCCTTCCATTTGAC | | MCL1 3´-UTR cloning |
| MCL1-NRV | GGGAAAGCGGCCGCTCATGTTCCGAGACTGAAGC | | MCL1 3´-UTR cloning |
| SPRY1-XFW | GGGAAACTCGAGCCTGTTTCCCACCTTCTCTTC | | SPRY1 3´-UTR cloning |
| SPRY1-NRV | GGGAAAGCGGCCGCCGTGTCACCTTATTTTCCGTTA | | SPRY1 3´-UTR cloning |
| SPRY1mut-1FW | AAAAAGCAACTGTTTAATTGCTTAATATTCGAATGTATTAAATCTGTCTCCAGTTAG | | miR-21 seed mutation in SPRY1 site 1 |
| SPRY1mut-1RV | CTAACTGGAGACAGATTTAATACATTCGAATATTAAGCAATTAAACAGTTGCTTTTT | | miR-21 seed mutation in SPRY1 site 1 |
| SPRY1mut-2FW | GATGATCTTCAGCAAGAGTGGACTGCCTTCGAGCACCTGGCTCCCACTTTCAACAAG | | miR-21 seed mutation in SPRY1 site 2 |
| SPRY1mut-2RV | CTTGTTGAAAGTGGGAGCCAGGTGCTCGAAGGCAGTCCACTCTTGCTGAAGATCATC | | miR-21 seed mutation in SPRY1 site 2 |

Bases targeted by site directed mutagenesis are underlined, “site 1” corresponds to chr4:124324121-124324128 and “site 2” to chr4:124323893-124323900
